# Supplementary material for: The elements of success in a comprehensive state-wide program to safely reduce the rate of preterm birth
Source: PLoS One. 2020 Jun 4;15(6):e0234033. doi: 10.1371/journal.pone.0234033 (PMC7272053; doi:10.1371/journal.pone.0234033)
Supplement: S1 Table — (PDF) [file pone.0234033.s001.pdf]

Table S1. Risk of preterm birth stratified by hospital level in unadjusted and adjusted models.

Year	N	n	(%)	OR	95% CI	p	aOR	95% CI	p
**Established tertiary**									
**2009**	5413	1036	19.1%	1.09	0.99-1.21	0.068	1.11	1.01-1.23	0.039
**2010**	5510	980	17.8%	1.00	0.91-1.10	0.996	1.03	0.93-1.14	0.594
**2011**	5405	974	18.0%	1.02	0.92-1.12	0.746	1.04	0.94-1.16	0.412
**2012**	5663	1068	18.9%	1.08	0.98-1.18	0.142	1.13	1.02-1.25	0.019
**2013**	5452	1121	20.6%	1.20	1.09-1.32	0.000	1.27	1.15-1.41	0.000
**2014**	5476	1054	19.3%	1.10	1.00-1.21	0.049	1.17	1.06-1.23	0.002
**2015**	5319	864	16.2%	0.90	0.81-0.99	0.034	0.96	0.87-1.07	0.489
**2016**	5304	933	17.6%	0.99	0.89-1.09	0.795	1.05	0.95-1.17	0.327
**2017**	5455	970	17.8%	1.000	Reference		1.00	Reference	
**Secondary/primary centres**									
**2009**	24820	1020	4.1%	0.77	0.71-0.84	0.000	0.76	0.70-0.82	0.000
**2010**	24846	1142	4.6%	0.87	0.80-0.94	0.000	0.86	0.79-0.93	0.000
**2011**	25821	1166	4.5%	0.85	0.78-0.92	0.000	0.85	0.78-0.92	0.000
**2012**	27208	1314	4.8%	0.91	0.84-0.99	0.020	0.92	0.85-0.99	0.031
**2013**	27945	1330	4.8%	0.90	0.83-0.97	0.007	0.91	0.84-0.98	0.017
**2014**	28525	1335	4.7%	0.88	0.82-0.95	0.002	0.89	0.82-0.96	0.004
**2015**	26429	1246	4.7%	0.89	0.82-0.96	0.004	0.90	0.83-0.98	0.010
**2016**	26823	1335	5.0%	0.94	0.87-1.02	0.124	0.95	0.88-1.03	0.209
**2017**	24987	1318	5.3%	1.00	Reference		1.00	Reference	
**State overall**									
**2009**	30233	2056	6.8%	0.89	0.84-0.95	0.000	0.93	0.88-0.99	0.023
**2010**	30356	2122	7.0%	0.92	0.86-0.97	0.004	0.96	0.90-1.02	0.193
**2011**	31226	2140	6.9%	0.90	0.85-0.95	0.000	0.93	0.88-0.99	0.020
**2012**	32871	2382	7.2%	0.95	0.90-1.01	0.100	0.98	0.93-1.04	0.558
**2013**	33397	2451	7.3%	0.97	0.91-1.02	0.233	1.00	0.94-1.06	0.878
**2014**	34115	2391	7.0%	0.92	0.87-0.97	0.004	0.94	0.89-0.999	0.047
**2015**	33944	2315	6.8%	0.89	0.84-0.95	0.000	0.92	0.87-0.98	0.007
**2016**	34854	2492	7.1%	0.94	0.89-0.99	0.031	0.96	0.91-1.02	0.203
**2017**	33437	2535	7.6%	1.00	Reference		1.00	Reference	

Adjusted logistic regression model included maternal characteristics known at the time of the first antenatal visit. Adjustments included maternal age (<20 or ≥35 years), maternal ethnicity (Caucasian, Indigenous and other ethnicities), smoking during pregnancy, low socioeconomic status, pre-existing diabetes, pre-existing hypertension, asthma, pre-existing other maternal conditions, *in vitro* fertilization, history of stillbirth(s), history of PTB and caesarean section in the preceding pregnancy.

OR=unadjusted odds ratio; aOR=adjusted odd ratio; CI=confidence interval, N=number of births, n=number of preterm births, (%) = PTB incidence rate

OR significantly lower than in 2017; OR significantly higher than in 2017
