# Supplementary material for: The elements of success in a comprehensive state-wide program to safely reduce the rate of preterm birth
Source: PLoS One. 2020 Jun 4;15(6):e0234033. doi: 10.1371/journal.pone.0234033 (PMC7272053; doi:10.1371/journal.pone.0234033)
Supplement: S2 Table — (PDF) [file pone.0234033.s002.pdf]

Table S2. Gestational age specific risk of preterm birth in the established tertiary level center in unadjusted and adjusted models.

GA /Year		N	n	(%)	OR	95% CI	p	aOR	95% CI	p
**20-27**	**2009**	5413	136	2.51%	1.19	0.93-1.53	0.171	1.22	0.94-1.58	0.141
	**2010**	5510	133	2.41%	1.13	0.88-1.45	0.358	1.17	0.90-1.52	0.236
	**2011**	5405	122	2.26%	1.06	0.82-1.37	0.681	1.11	0.85-1.45	0.450
	**2012**	5663	127	2.24%	1.06	0.82-1.37	0.656	1.14	0.88-1.49	0.324
	**2013**	5452	119	2.18%	1.05	0.81-1.36	0.694	1.15	0.88-1.51	0.301
	**2014**	5476	135	2.47%	1.17	0.91-1.50	0.220	1.28	0.99-1.67	0.059
	**2015**	5319	113	2.12%	0.97	0.75-1.26	0.834	1.08	0.82-1.42	0.580
	**2016**	5304	134	2.53%	1.18	0.91-1.51	0.208	1.29	1.00-1.67	0.054
	**2017**	5455	117	2.14%	1.00			1.00		
**28-31**	**2009**	5413	162	2.99%	1.22	0.97-1.54	0.092	1.27	1.00-1.61	0.055
	**2010**	5510	150	2.72%	1.09	0.86-1.38	0.464	1.16	0.91-1.48	0.245
	**2011**	5405	139	2.57%	1.04	0.81-1.32	0.782	1.09	0.85-1.40	0.480
	**2012**	5663	169	2.98%	1.21	0.96-1.53	0.099	1.32	1.04-1.68	0.022
	**2013**	5452	159	2.92%	1.21	0.96-1.53	0.107	1.34	1.05-1.70	0.019
	**2014**	5476	169	3.09%	1.26	1.00-1.59	0.048	1.39	1.10-1.76	0.007
	**2015**	5319	134	2.52%	0.99	0.78-1.26	0.948	1.12	0.87-1.43	0.392
	**2016**	5304	157	2.96%	1.19	0.94-1.50	0.155	1.31	1.03-1.66	0.029
	**2017**	5455	136	2.49%	1.00			1.00		
**32-36**	**2009**	5413	738	13.63%	1.06	0.94-1.18	0.347	1.07	0.95-1.20	0.271
	**2010**	5510	697	12.65%	0.96	0.86-1.08	0.504	0.98	0.87-1.10	0.756
	**2011**	5405	713	13.19%	1.01	0.90-1.13	0.909	1.03	0.91-1.15	0.678
	**2012**	5663	772	13.63%	1.05	0.94-1.17	0.375	1.09	0.97-1.22	0.134
	**2013**	5452	843	15.46%	1.22	1.09-1.36	0.000	1.28	1.14-1.43	0.000
	**2014**	5476	750	13.70%	1.06	0.95-1.19	0.294	1.11	0.99-1.25	0.067
	**2015**	5319	617	11.60%	0.87	0.77-0.97	0.015	0.92	0.81-1.03	0.156
	**2016**	5304	642	12.10%	0.92	0.82-1.03	0.146	0.97	0.86-1.09	0.580
	**2017**	5455	717	13.14%	1.000			1.00		

Adjusted logistic regression model included maternal characteristics known at the time of the first antenatal visit. Adjustments included maternal age (<20 or ≥35 years), maternal ethnicity (Caucasian, Indigenous and other ethnicities), smoking during pregnancy, low socioeconomic status, pre-existing diabetes, pre-existing hypertension, asthma, pre-existing other maternal conditions, *in vitro* fertilization, history of stillbirth(s), history of PTB and caesarean section in the preceding pregnancy.

OR=unadjusted odds ratio; aOR=adjusted odds ratio; CI=confidence interval, N=number of births, n=number of preterm births, (%) = PTB incidence rate

OR significantly lower than in 2017; OR significantly higher than in 2017
