# Supplementary material for: The elements of success in a comprehensive state-wide program to safely reduce the rate of preterm birth
Source: PLoS One. 2020 Jun 4;15(6):e0234033. doi: 10.1371/journal.pone.0234033 (PMC7272053; doi:10.1371/journal.pone.0234033)
Supplement: S3 Table — (PDF) [file pone.0234033.s003.pdf]

Table S3. Gestational age specific risk of preterm birth in the secondary/primary level centres in unadjusted and adjusted models.

GA /Year		N	n	(%)	OR	95% CI	p	aOR	95% CI	p
**20-27**	**2009**	24820	38	0.15%	0.90	0.58-1.40	0.637	0.89	0.57-1.40	0.613
	**2010**	24846	36	0.14%	0.86	0.55-1.34	0.494	0.86	0.55-1.36	0.522
	**2011**	25821	47	0.18%	1.07	0.71-1.63	0.736	1.10	0.72-1.68	0.667
	**2012**	27208	47	0.17%	1.02	0.67-1.55	0.915	1.10	0.72-1.69	0.649
	**2013**	27945	49	0.18%	1.04	0.69-1.57	0.861	1.13	0.74-1.72	0.571
	**2014**	28525	45	0.16%	0.93	0.61-1.42	0.746	1.00	0.65-1.52	0.988
	**2015**	26429	37	0.14%	0.83	0.53-1.29	0.403	0.90	0.58-1.41	0.654
	**2016**	26823	44	0.16%	0.97	0.64-1.49	0.899	1.06	0.69-1.62	0.803
	**2017**	24987	42	0.17%	1.00			1.00		
**28-31**	**2009**	24820	37	0.15%	1.08	0.68-1.73	0.740	1.02	0.64-1.65	0.923
	**2010**	24846	23	0.09%	0.68	0.40-1.15	0.146	0.66	0.39-1.13	0.130
	**2011**	25821	28	0.11%	0.79	0.48-1.30	0.357	0.78	0.47-1.30	0.344
	**2012**	27208	28	0.10%	0.75	0.46-1.24	0.266	0.76	0.46-1.26	0.285
	**2013**	27945	30	0.11%	0.79	0.48-1.28	0.333	0.79	0.48-1.30	0.359
	**2014**	28525	26	0.09%	0.67	0.40-1.11	0.118	0.68	0.40-1.13	0.134
	**2015**	26429	25	0.09%	0.69	0.41-1.16	0.161	0.71	0.42-1.19	0.188
	**2016**	26823	43	0.16%	1.17	0.75-1.84	0.484	1.21	0.77-1.90	0.415
	**2017**	24987	34	0.14%	1.00			1.00		
**32-36**	**2009**	24820	945	3.81%	0.76	0.69-0.83	0.000	0.74	0.68-0.81	0.000
	**2010**	24846	1083	4.36%	0.87	0.80-0.95	0.001	0.86	0.79-0.94	0.001
	**2011**	25821	1091	4.23%	0.84	0.78-0.92	0.000	0.84	0.77-0.91	0.000
	**2012**	27208	1239	4.55%	0.91	0.84-0.99	0.025	0.91	0.84-0.99	0.031
	**2013**	27945	1251	4.48%	0.90	0.83-0.97	0.007	0.90	0.83-0.98	0.014
	**2014**	28525	1264	4.43%	0.89	0.82-0.96	0.003	0.89	0.82-0.97	0.006
	**2015**	26429	1184	4.48%	0.90	0.83-0.97	0.008	0.91	0.83-0.98	0.018
	**2016**	26823	1248	4.65%	0.93	0.86-1.01	0.092	0.94	0.87-1.02	0.137
	**2017**	24987	1242	4.97%	1.00			1.00		

Adjusted logistic regression model included maternal characteristics known at the time of the first antenatal visit. Adjustments included maternal age (<20 or ≥35 years), maternal ethnicity (Caucasian, Indigenous and other ethnicities), smoking during pregnancy, low socioeconomic status, pre-existing diabetes, pre-existing hypertension, asthma, pre-existing other maternal conditions, *in vitro* fertilization, history of stillbirth(s), history of PTB and caesarean section in the preceding pregnancy.

OR=unadjusted odds ratio; aOR=adjusted odds ratio; CI=confidence interval, N=number of births, n=number of preterm births, (%) = PTB incidence rate; OR significantly lower than in 2017
