# Supplementary material for: The elements of success in a comprehensive state-wide program to safely reduce the rate of preterm birth
Source: PLoS One. 2020 Jun 4;15(6):e0234033. doi: 10.1371/journal.pone.0234033 (PMC7272053; doi:10.1371/journal.pone.0234033)
Supplement: S4 Table — (PDF) [file pone.0234033.s004.pdf]

Table S4. Gestational age specific risk of preterm birth overall in unadjusted and adjusted models.

GA /Year		N	n	(%)	OR	95% CI	p	aOR	95% CI	p
**20-27**	**2009**	30233	174	0.58%	1.18	0.95-1.46	0.135	1.35	1.08-1.68	0.008
	**2010**	30356	169	0.56%	1.14	0.92-1.42	0.229	1.30	1.04-1.62	0.021
	**2011**	31226	169	0.54%	1.11	0.89-1.38	0.351	1.24	1.00-1.55	0.056
	**2012**	32871	174	0.53%	1.09	0.88-1.35	0.438	1.21	0.97-1.51	0.089
	**2013**	33397	168	0.50%	1.04	0.83-1.29	0.752	1.13	0.91-1.41	0.266
	**2014**	34115	180	0.53%	1.08	0.88-1.34	0.466	1.17	0.94-1.45	0.153
	**2015**	33944	156	0.46%	0.94	0.76-1.17	0.588	1.03	0.82-1.28	0.824
	**2016**	34854	183	0.53%	1.08	0.87-1.33	0.484	1.16	0.93-1.43	0.188
	**2017**	33437	162	0.48%	1.00			1.00		
**28-31**	**2009**	30233	199	0.66%	1.14	0.93-1.39	0.207	1.28	1.04-1.56	0.020
	**2010**	30356	173	0.57%	0.99	0.80-1.21	0.895	1.10	0.90-1.36	0.356
	**2011**	31226	167	0.53%	0.92	0.75-1.14	0.457	1.01	0.82-1.25	0.914
	**2012**	32871	197	0.60%	1.04	0.85-1.27	0.700	1.12	0.91-1.37	0.280
	**2013**	33397	189	0.57%	0.98	0.80-1.20	0.867	1.04	0.85-1.28	0.683
	**2014**	34115	195	0.57%	0.99	0.81-1.21	0.916	1.04	0.85-1.28	0.680
	**2015**	33944	168	0.49%	0.86	0.70-1.05	0.139	0.91	0.74-1.12	0.380
	**2016**	34854	205	0.59%	1.02	0.84-1.24	0.848	1.07	0.88-1.31	0.500
	**2017**	33437	192	0.57%	1.00			1.00		
**32-36**	**2009**	30233	1683	5.57%	0.85	0.79-0.90	0.000	0.87	0.82-0.93	0.000
	**2010**	30356	1780	5.86%	0.89	0.84-0.95	0.001	0.92	0.87-0.99	0.020
	**2011**	31226	1804	5.78%	0.88	0.82-0.94	0.000	0.90	0.84-0.96	0.002
	**2012**	32871	2011	6.12%	0.94	0.88-1.00	0.034	0.96	0.90-1.02	0.155
	**2013**	33397	2094	6.27%	0.96	0.90-1.02	0.183	0.98	0.92-1.05	0.554
	**2014**	34115	2016	5.91%	0.90	0.85-0.96	0.001	0.92	0.86-0.98	0.008
	**2015**	33944	1991	5.87%	0.89	0.84-0.95	0.000	0.92	0.86-0.98	0.006
	**2016**	34854	2104	6.04%	0.92	0.87-0.98	0.009	0.94	0.88-1.00	0.051
	**2017**	33437	2181	6.52%	1.00			1.00		

Adjusted nominal logistic regression model included maternal characteristics known at the time of the first antenatal visit. Adjustments included maternal age (<20 or ≥35 years), maternal ethnicity (Caucasian, Indigenous and other ethnicities), smoking during pregnancy, low socioeconomic status, pre-existing diabetes, pre-existing hypertension, asthma, pre-existing other maternal conditions, *in vitro* fertilization, history of stillbirth(s), history of PTB and caesarean section in the preceding pregnancy.

OR=unadjusted odds ratio; aOR=adjusted odds ratio; CI=confidence interval, N=number of births, n=number of preterm births, (%) = PTB incidence rate

OR significantly lower than in 2017; OR significantly higher than in 2017
