# Supplementary material for: The elements of success in a comprehensive state-wide program to safely reduce the rate of preterm birth
Source: PLoS One. 2020 Jun 4;15(6):e0234033. doi: 10.1371/journal.pone.0234033 (PMC7272053; doi:10.1371/journal.pone.0234033)
Supplement: S5 Table — (PDF) [file pone.0234033.s005.pdf]

Table S5. Unadjusted risk of preterm birth for maternal characteristics known at the time of the first antenatal visit in singleton pregnancies in years 2009-207.

Characteristics		PTB (%)	OR	95% CI	p
**Maternal demographics**					
Maternal age	20-34	6.7%	1.00		
	<20	10.1%	1.56	1.46-1.66	<0.001
	≥35	7.9%	1.19	1.15-1.23	<0.001
Ethnicity	Caucasian	6.7%	1.00	Reference	
	Indigenous	14.0%	2.28	2.17-2.40	<0.001
	Other	6.9%	1.04	1.04-1.08	0.030
Parity	1-4	6.7%	1.00	Reference	
	≥5	12.6%	2.02	1.85-2.21	<0.001
	0	7.4%	1.12	1.09-1.15	<0.001
Smoking during pregnancy	No	6.5%	1.00	Reference	
	Yes	11.6%	1.88	1.18-1.95	<0.001
Low socioeconomic status	No	6.6%	1.00	Reference	
	Yes	7.8%	1.21	1.17-1.24	<0.001
**Medical conditions**					
Pre-existing diabetes	No	6.9%	1.00	Reference	
	Yes	30.6%	5.92	5.40-6.49	<0.001
Pre-existing hypertension	No	7.0%	1.00	Reference	
	Yes	19.2%	3.19	2.92-3.48	<0.001
Asthma	No	7.0%	1.00	Reference	
	Yes	7.8%	1.13	1.08-1.18	<0.001
Other pre-existing conditions	No	6.4%	1.00	Reference	
	Yes	8.6%	1.39	1.35-1.43	<0.001
**Obstetric history**					
Previous PTB	No	4.5%	1.00	Reference	
	Yes	26.1%	7.46	7.01-7.94	<0.001
	unknown	6.8%	1.55	1.48-1.62	<0.001
Previous stillbirths	None	6.9%	1.00	Reference	
	1	18.3%	3.01	2.76-3.28	<0.001
	≥2	22.7%	3.97	2.97-5.23	<0.001
CS at last birth	No	6.9%	1.00	Reference	
	Yes	8.2%	1.21	1.17-1.25	<0.001
IVF conception	No	7.0%	1.00	Reference	
	Yes	10.1%	1.49	1.39-1.60	<0.001
**High risk at the first antenatal visit**	No	5.5%	1.00	Reference	
	Yes	13.7%	2.71	2.63-2.79	<0.001

*nulliparous and parous women combined after predictive models for nulliparous (n=125407) and parous (n=169026) women were constructed separately.

OR=unadjusted odds ratio; CI=confidence interval, PTB (%) = PTB incidence rate
