# Supplementary material for: The elements of success in a comprehensive state-wide program to safely reduce the rate of preterm birth
Source: PLoS One. 2020 Jun 4;15(6):e0234033. doi: 10.1371/journal.pone.0234033 (PMC7272053; doi:10.1371/journal.pone.0234033)
Supplement: S6 Table — (PDF) [file pone.0234033.s006.pdf]

Table S6. Risk of preterm birth in low risk singleton pregnancies stratified by hospital level in unadjusted and adjusted models.

Year	N	n	(%)	OR	95% CI	p	aOR	95% CI	p
**Established tertiary**									
**2009**	3930	643	16.4%	1.34	1.17-1.52	0.000	1.31	1.15-1.50	0.000
**2010**	3979	593	14.9%	1.20	1.05-1.36	0.008	1.19	1.04-1.36	0.012
**2011**	3804	558	14.7%	1.17	1.03-1.34	0.019	1.19	1.03-1.36	0.015
**2012**	4001	582	14.5%	1.16	1.02-1.33	0.026	1.19	1.04-1.36	0.013
**2013**	3883	626	16.1%	1.31	1.15-1.50	0.000	1.36	1.19-1.55	0.000
**2014**	3888	591	15.2%	1.22	1.07-1.40	0.003	1.26	1.10-1.44	0.001
**2015**	3835	460	12.0%	0.93	0.81-1.07	0.307	0.96	0.83-1.11	0.578
**2016**	3710	485	13.1%	1.03	0.89-1.18	0.711	1.05	0.91-1.21	0.495
**2017**	3521	450	12.8%	1.00	Reference		1.00	Reference	
**Secondary/primary centres**									
**2009**	20644	729	3.5%	0.85	0.77-0.94	0.002	0.84	0.75-0.93	0.001
**2010**	20868	840	4.0%	0.98	0.89-1.08	0.625	0.96	0.87-1.06	0.393
**2011**	21554	855	4.0%	0.96	0.87-1.06	0.424	0.95	0.86-1.05	0.288
**2012**	22629	915	4.0%	0.98	0.89-1.08	0.687	0.97	0.88-1.07	0.562
**2013**	23253	933	4.0%	0.97	0.88-1.07	0.568	0.97	0.88-1.07	0.547
**2014**	23576	908	3.9%	0.93	0.85-1.03	0.150	0.93	0.84-1.02	0.119
**2015**	21999	855	3.9%	0.94	0.85-1.04	0.220	0.94	0.85-1.04	0.212
**2016**	22182	878	4.0%	0.96	0.87-1.06	0.395	0.96	0.87-1.06	0.383
**2017**	20289	836	4.1%	1.00	Reference		1.00	Reference	
**State overall**									
**2009**	24574	1372	5.6%	1.02	0.95-1.10	0.616	1.06	0.98-1.15	0.132
**2010**	24847	1433	5.8%	1.06	0.98-1.14	0.161	1.09	1.01-1.18	0.023
**2011**	25358	1413	5.6%	1.02	0.94-1.10	0.651	1.04	0.97-1.13	0.278
**2012**	26630	1497	5.6%	1.03	0.95-1.11	0.481	1.04	0.97-1.12	0.271
**2013**	27136	1559	5.7%	1.05	0.98-1.13	0.186	1.06	0.99-1.15	0.102
**2014**	27565	1500	5.4%	0.99	0.92-1.07	0.840	1.00	0.92-1.07	0.891
**2015**	27676	1457	5.3%	0.96	0.89-1.03	0.265	0.97	0.90-1.04	0.369
**2016**	28125	1515	5.4%	0.98	0.91-1.06	0.627	0.98	0.91-1.06	0.665
**2017**	26254	1439	5.5%	1.00	Reference		1.00	Reference	

Adjusted logistic regression model included maternal characteristics known at the time of the first antenatal visit. Adjustments included maternal age (<20 or ≥35 years), maternal ethnicity (Caucasian, Indigenous and other ethnicities), smoking during pregnancy, low socioeconomic status, pre-existing diabetes, pre-existing hypertension, asthma, pre-existing other maternal conditions, *in vitro* fertilization, history of stillbirth(s), history of PTB and caesarean section in the preceding pregnancy.

OR=unadjusted odds ratio; aOR=adjusted odds ratio; CI=confidence interval, N=number of births, n=number of preterm births, (%) = PTB incidence rate

OR significantly lower than in 2017; OR significantly higher than in 2017
