# Supplementary material for: The elements of success in a comprehensive state-wide program to safely reduce the rate of preterm birth
Source: PLoS One. 2020 Jun 4;15(6):e0234033. doi: 10.1371/journal.pone.0234033 (PMC7272053; doi:10.1371/journal.pone.0234033)
Supplement: S7 Table — (PDF) [file pone.0234033.s007.pdf]

Table S7. Risk of preterm birth in high risk singleton pregnancies stratified by hospital level in unadjusted and adjusted models.

Year	N	n	(%)	OR	95% CI	p	aOR	95% CI	p
**Established tertiary**									
**2009**	1483	393	26.5%	0.98	0.84-1.14	0.800	0.92	0.79-1.09	0.344
**2010**	1531	387	25.3%	0.92	0.79-1.07	0.284	0.87	0.74-1.03	0.099
**2011**	1601	416	26.0%	0.96	0.82-1.11	0.544	0.90	0.77-1.06	0.203
**2012**	1662	486	29.2%	1.12	0.97-1.30	0.117	1.09	0.93-1.27	0.292
**2013**	1569	495	31.5%	1.25	1.08-1.45	0.003	1.21	1.03-1.41	0.018
**2014**	1588	463	29.2%	1.12	0.97-1.30	0.135	1.08	0.92-1.26	0.344
**2015**	1484	404	27.2%	1.02	0.87-1.18	0.826	0.98	0.84-1.16	0.842
**2016**	1594	448	28.1%	1.06	0.92-1.23	0.420	1.05	0.90-1.22	0.555
**2017**	1934	520	26.9%	1.00	Reference		1.00	Reference	
**Secondary/primary centres**									
**2009**	4176	291	7.0%	0.66	0.56-0.76	0.000	0.65	0.56-0.76	0.000
**2010**	3978	302	7.6%	0.72	0.62-0.84	0.000	0.71	0.61-0.83	0.000
**2011**	4267	311	7.3%	0.69	0.59-0.80	0.000	0.67	0.58-0.79	0.000
**2012**	4579	399	8.7%	0.84	0.73-0.96	0.011	0.82	0.71-0.94	0.005
**2013**	4692	397	8.5%	0.81	0.70-0.93	0.003	0.80	0.69-0.92	0.002
**2014**	4949	427	8.6%	0.83	0.72-0.95	0.006	0.82	0.71-0.94	0.004
**2015**	4430	391	8.8%	0.85	0.74-0.97	0.020	0.83	0.72-0.95	0.008
**2016**	4641	457	9.8%	0.96	0.84-1.09	0.507	0.93	0.81-1.07	0.306
**2017**	4698	482	10.3%	1.00	Reference		1.00	Reference	
**State overall**									
**2009**	5659	684	12.1%	0.76	0.69-0.85	0.000	0.76	0.68-0.85	0.000
**2010**	5509	689	12.5%	0.79	0.72-0.88	0.000	0.78	0.70-0.87	0.000
**2011**	5868	727	12.4%	0.79	0.71-0.87	0.000	0.77	0.69-0.85	0.000
**2012**	6241	885	14.2%	0.92	0.83-1.01	0.079	0.89	0.81-0.98	0.021
**2013**	6261	892	14.2%	0.92	0.84-1.02	0.099	0.89	0.81-0.99	0.025
**2014**	6550	891	13.6%	0.87	0.80-0.96	0.006	0.85	0.77-0.94	0.001
**2015**	6268	858	13.7%	0.88	0.80-0.97	0.010	0.85	0.77-0.94	0.001
**2016**	6729	977	14.5%	0.94	0.86-1.04	0.221	0.92	0.83-1.01	0.076
**2017**	7183	1096	15.3%	1.00	Reference		1.00	Reference	

Adjusted logistic regression model included maternal characteristics known at the time of the first antenatal visit. Adjustments included maternal age (<20 or ≥35 years), maternal ethnicity (Caucasian, Indigenous and other ethnicities), smoking during pregnancy, low socioeconomic status, pre-existing diabetes, pre-existing hypertension, asthma, pre-existing other maternal conditions, *in vitro* fertilization, history of stillbirth(s), history of PTB and caesarean section in the preceding pregnancy.

OR=unadjusted odds ratio; aOR=adjusted odds ratio; CI=confidence interval, N=number of births, n=number of preterm births, (%) = PTB incidence rate;

OR significantly lower than in 2017; OR significantly higher than in 2017
