# Supplementary material for: The elements of success in a comprehensive state-wide program to safely reduce the rate of preterm birth
Source: PLoS One. 2020 Jun 4;15(6):e0234033. doi: 10.1371/journal.pone.0234033 (PMC7272053; doi:10.1371/journal.pone.0234033)
Supplement: S8 Table — (PDF) [file pone.0234033.s008.pdf]

Table S8. Gestational age specific risk of preterm birth in low risk singleton pregnancies at tertiary level center in unadjusted and adjusted models.

GA /Year		N	n	(%)	OR	95% CI	p	aOR	95% CI	p
**20-27**	**2009**	3930	93	2.37%	1.36	0.98-1.87	0.063	1.20	0.87-1.67	0.270
	**2010**	3979	69	1.73%	0.98	0.69-1.38	0.898	0.89	0.63-1.25	0.493
	**2011**	3804	70	1.84%	1.04	0.74-1.46	0.845	0.97	0.69-1.38	0.876
	**2012**	4001	83	2.07%	1.17	0.84-1.62	0.364	1.13	0.81-1.58	0.461
	**2013**	3883	71	1.83%	1.05	0.74-1.47	0.796	1.05	0.74-1.48	0.793
	**2014**	3888	86	2.21%	1.25	0.90-1.74	0.179	1.24	0.89-1.73	0.197
	**2015**	3835	80	2.09%	1.14	0.82-1.59	0.448	1.14	0.82-1.60	0.443
	**2016**	3710	78	2.10%	1.16	0.83-1.62	0.383	1.17	0.83-1.63	0.367
	**2017**	3521	64	1.82%	1.00			1.00		
**28-31**	**2009**	3930	110	2.80%	1.87	1.35-2.59	0.000	1.91	1.37-2.68	0.000
	**2010**	3979	102	2.56%	1.68	1.21-2.34	0.002	1.75	1.25-2.46	0.001
	**2011**	3804	74	1.95%	1.27	0.90-1.81	0.180	1.35	0.94-1.94	0.101
	**2012**	4001	89	2.22%	1.45	1.04-2.04	0.031	1.56	1.11-2.21	0.012
	**2013**	3883	81	2.09%	1.39	0.98-1.96	0.063	1.52	1.07-2.17	0.019
	**2014**	3888	94	2.42%	1.59	1.14-2.23	0.007	1.73	1.23-2.44	0.002
	**2015**	3835	69	1.80%	1.14	0.80-1.63	0.468	1.24	0.86-1.79	0.247
	**2016**	3710	85	2.29%	1.47	1.05-2.07	0.027	1.56	1.10-2.21	0.012
	**2017**	3521	55	1.56%	1.00			1.00		
**32-36**	**2009**	3930	440	11.26	1.24	1.07-1.44	0.005	1.24	1.06-1.44	0.007
	**2010**	3979	422	10.56	1.16	0.99-1.35	0.061	1.16	1.00-1.36	0.056
	**2011**	3804	414	10.86	1.18	1.02-1.38	0.031	1.20	1.03-1.40	0.021
	**2012**	4001	410	10.29	1.11	0.96-1.30	0.171	1.14	0.97-1.33	0.110
	**2013**	3883	474	12.25	1.35	1.16-1.57	0.000	1.39	1.20-1.62	0.000
	**2014**	3888	411	10.53	1.16	0.99-1.35	0.062	1.18	1.01-1.38	0.035
	**2015**	3835	311	8.11%	0.86	0.73-1.01	0.058	0.88	0.75-1.04	0.128
	**2016**	3710	322	8.91%	0.93	0.79-1.09	0.352	0.94	0.80-1.11	0.487
	**2017**	3521	331	9.28%	1.00			1.00		

Adjusted nominal logistic regression model included maternal characteristics known at the time of the first antenatal visit. Adjustments included maternal age (<20 or ≥35 years), maternal ethnicity (Caucasian, Indigenous and other ethnicities), smoking during pregnancy, low socioeconomic status, pre-existing diabetes, pre-existing hypertension, asthma, pre-existing other maternal conditions, *in vitro* fertilization, history of stillbirth(s) , history of PTB and caesarean section in the preceding pregnancy.

OR=unadjusted odds ratio; aOR=adjusted odds ratio; CI=confidence interval, N=number of births, n=number of preterm births, (%) = PTB incidence rate; OR significantly higher than in 2017
