# Supplementary material for: The elements of success in a comprehensive state-wide program to safely reduce the rate of preterm birth
Source: PLoS One. 2020 Jun 4;15(6):e0234033. doi: 10.1371/journal.pone.0234033 (PMC7272053; doi:10.1371/journal.pone.0234033)
Supplement: S9 Table — (PDF) [file pone.0234033.s009.pdf]

Table 9. Gestational age specific risk of preterm birth in low risk singleton pregnancies in secondary/primary level centers, in unadjusted and adjusted models.

		N	n	(%)	OR	95% CI	p	aOR	95% CI	p
**20-27**	**2009**	20644	22	0.11%	0.83	0.47-1.46	0.511	0.77	0.43-1.37	0.370
	**2010**	20868	20	0.10%	0.75	0.42-1.34	0.327	0.69	0.38-1.25	0.224
	**2011**	21554	27	0.13%	0.98	0.57-1.67	0.929	0.93	0.54-1.61	0.806
	**2012**	22629	28	0.12%	0.97	0.57-1.65	0.895	0.95	0.56-1.64	0.864
	**2013**	23253	32	0.14%	1.07	0.64-1.80	0.791	1.08	0.64-1.83	0.766
	**2014**	23576	30	0.13%	0.99	0.59-1.68	0.971	1.00	0.59-1.69	0.990
	**2015**	21999	28	0.13%	0.99	0.58-1.69	0.973	1.01	0.59-1.72	0.982
	**2016**	22182	27	0.12%	0.95	0.55-1.63	0.847	0.98	0.57-1.68	0.931
	**2017**	20289	26	0.13%	1.00			1.00		
**28-31**	**2009**	20644	24	0.12%	0.94	0.54-1.64	0.822	0.87	0.49-1.53	0.627
	**2010**	20868	15	0.07%	0.58	0.31-1.11	0.098	0.54	0.29-1.04	0.065
	**2011**	21554	20	0.09%	0.75	0.42-1.35	0.342	0.70	0.39-1.27	0.246
	**2012**	22629	13	0.06%	0.47	0.24-0.91	0.026	0.44	0.23-0.87	0.018
	**2013**	23253	19	0.08%	0.66	0.37-1.20	0.176	0.64	0.35-1.16	0.141
	**2014**	23576	14	0.06%	0.48	0.25-0.93	0.028	0.47	0.24-0.90	0.022
	**2015**	21999	15	0.07%	0.55	0.29-1.05	0.069	0.54	0.28-1.02	0.057
	**2016**	22182	24	0.11%	0.88	0.50-1.54	0.645	0.86	0.49-1.51	0.601
	**2017**	20289	25	0.12%	1.00			1.00		
**32-36**	**2009**	20644	683	3.31%	0.85	0.77-0.94	0.002	0.84	0.75-0.93	0.001
	**2010**	20868	805	3.86%	1.00	0.90-1.10	0.938	0.98	0.89-1.09	0.703
	**2011**	21554	808	3.75%	0.97	0.88-1.07	0.516	0.96	0.87-1.06	0.386
	**2012**	22629	874	3.86%	1.00	0.90-1.10	0.959	0.99	0.90-1.09	0.836
	**2013**	23253	882	3.79%	0.98	0.89-1.08	0.675	0.98	0.89-1.08	0.658
	**2014**	23576	864	3.66%	0.95	0.86-1.04	0.256	0.94	0.85-1.04	0.210
	**2015**	21999	812	3.69%	0.95	0.86-1.05	0.332	0.95	0.86-1.05	0.322
	**2016**	22182	827	3.73%	0.96	0.87-1.06	0.445	0.96	0.87-1.06	0.424
	**2017**	20289	785	3.87%	1.00			1.00		

Adjusted nominal logistic regression model included maternal characteristics known at the time of the first antenatal visit. Adjustments included maternal age (<20 or ≥35 years), maternal ethnicity (Caucasian, Indigenous and other ethnicities), smoking during pregnancy, low socioeconomic status, pre-existing diabetes, pre-existing hypertension, asthma, pre-existing other maternal conditions, *in vitro* fertilization, history of stillbirth(s), history of PTB and caesarean section in the preceding pregnancy.

OR=unadjusted odds ratio; aOR=adjusted odds ratio; CI=confidence interval, N=number of births, n=number of preterm births, (%) = PTB incidence rate

OR significantly lower than in 2017; OR significantly higher than in 2017
