# Supplementary material for: The elements of success in a comprehensive state-wide program to safely reduce the rate of preterm birth
Source: PLoS One. 2020 Jun 4;15(6):e0234033. doi: 10.1371/journal.pone.0234033 (PMC7272053; doi:10.1371/journal.pone.0234033)
Supplement: S10 Table — (PDF) [file pone.0234033.s010.pdf]

Table S10. Gestational age specific risk of preterm birth in low risk singleton pregnancies state-wide in unadjusted and adjusted models.

		N	n	(%)	OR	95% CI	p	aOR	95% CI	p
**20-27**	**2009**	24574	115	0.47%	1.32	1.01-1.74	0.045	1.40	1.06-1.85	0.017
	**2010**	24847	89	0.36%	1.01	0.76-1.36	0.924	1.06	0.79-1.43	0.691
	**2011**	25358	97	0.38%	1.08	0.81-1.44	0.593	1.12	0.84-1.49	0.438
	**2012**	26630	111	0.42%	1.18	0.89-1.55	0.244	1.21	0.92-1.60	0.172
	**2013**	27136	103	0.38%	1.08	0.81-1.42	0.616	1.09	0.82-1.44	0.558
	**2014**	27565	116	0.42%	1.19	0.90-1.56	0.218	1.19	0.91-1.57	0.205
	**2015**	27676	112	0.40%	1.14	0.87-1.50	0.352	1.15	0.87-1.52	0.315
	**2016**	28125	108	0.38%	1.08	0.82-1.43	0.574	1.09	0.83-1.44	0.534
	**2017**	26254	93	0.35%	1.00			1.00		
**28-31**	**2009**	24574	134	0.55%	1.53	1.17-1.99	0.002	1.71	1.31-2.23	0.000
	**2010**	24847	117	0.47%	1.32	1.01-1.73	0.046	1.46	1.11-1.92	0.008
	**2011**	25358	94	0.37%	1.04	0.78-1.38	0.807	1.12	0.84-1.49	0.462
	**2012**	26630	102	0.38%	1.07	0.81-1.42	0.630	1.12	0.85-1.49	0.421
	**2013**	27136	100	0.37%	1.03	0.78-1.37	0.826	1.07	0.80-1.42	0.656
	**2014**	27565	108	0.39%	1.09	0.83-1.44	0.526	1.11	0.84-1.47	0.463
	**2015**	27676	89	0.32%	0.90	0.67-1.20	0.459	0.92	0.69-1.23	0.558
	**2016**	28125	111	0.39%	1.10	0.84-1.45	0.492	1.11	0.84-1.47	0.450
	**2017**	26254	94	0.36%	1.00			1.00		
**32-36**	**2009**	24574	1123	4.57%	0.96	0.88-1.04	0.324	0.99	0.91-1.08	0.825
	**2010**	24847	1227	4.94%	1.04	0.96-1.13	0.357	1.07	0.99-1.16	0.112
	**2011**	25358	1222	4.82%	1.01	0.93-1.10	0.781	1.03	0.95-1.12	0.451
	**2012**	26630	1284	4.82%	1.01	0.94-1.10	0.759	1.02	0.95-1.11	0.558
	**2013**	27136	1356	5.00%	1.05	0.97-1.14	0.218	1.06	0.98-1.15	0.138
	**2014**	27565	1276	4.63%	0.97	0.90-1.05	0.459	0.97	0.90-1.05	0.480
	**2015**	27676	1256	4.54%	0.95	0.88-1.03	0.205	0.96	0.88-1.04	0.271
	**2016**	28125	1296	4.61%	0.97	0.89-1.05	0.385	0.97	0.89-1.05	0.397
	**2017**	26254	1252	4.77%	1.00			1.00		

Adjusted nominal logistic regression model included maternal characteristics known at the time of the first antenatal visit. Adjustments included maternal age (<20 or ≥35 years), maternal ethnicity (Caucasian, Indigenous and other ethnicities), smoking during pregnancy, low socioeconomic status, pre-existing diabetes, pre-existing hypertension,asthma, pre-existing other maternal conditions, *in vitro* fertilization, history of stillbirth(s), history of PTB and caesarean section in the preceding pregnancy.

OR=unadjusted odds ratio; aOR=adjusted odds ratio; CI=confidence interval, N=number of births, n=number of preterm births, (%) = PTB incidence rate; OR significantly higher than in 2017
