# Supplementary material for: The elements of success in a comprehensive state-wide program to safely reduce the rate of preterm birth
Source: PLoS One. 2020 Jun 4;15(6):e0234033. doi: 10.1371/journal.pone.0234033 (PMC7272053; doi:10.1371/journal.pone.0234033)
Supplement: S11 Table — (PDF) [file pone.0234033.s011.pdf]

Table S11. Gestational age specific risk of preterm birth in high risk singleton pregnancies at tertiary level center in unadjusted and adjusted models.

GA /Year		N	n	(%)	OR	95% CI	p	aOR	95% CI	p
**20-27**	**2009**	1483	43	2.90%	1.05	0.70-1.59	0.807	1.17	0.75-1.81	0.488
	**2010**	1531	64	4.18%	1.49	1.03-2.17	0.035	1.67	1.12-2.50	0.012
	**2011**	1601	52	3.25%	1.17	0.79-1.73	0.429	1.30	0.86-1.97	0.220
	**2012**	1662	44	2.65%	1.00	0.66-1.50	0.993	1.13	0.73-1.74	0.588
	**2013**	1569	48	3.06%	1.19	0.80-1.78	0.387	1.33	0.87-2.03	0.189
	**2014**	1588	49	3.09%	1.16	0.78-1.73	0.458	1.32	0.87-2.00	0.199
	**2015**	1484	33	2.22%	0.82	0.52-1.27	0.365	0.93	0.59-1.48	0.766
	**2016**	1594	56	3.51%	1.30	0.89-1.91	0.175	1.45	0.98-2.16	0.064
	**2017**	1934	53	2.74%	1.00			1.00		
**28-31**	**2009**	1483	52	3.51%	0.83	0.58-1.19	0.315	0.77	0.53-1.12	0.178
	**2010**	1531	48	3.14%	0.73	0.51-1.06	0.095	0.70	0.48-1.02	0.063
	**2011**	1601	65	4.06%	0.96	0.69-1.34	0.800	0.90	0.63-1.28	0.544
	**2012**	1662	80	4.81%	1.19	0.86-1.63	0.290	1.15	0.82-1.61	0.412
	**2013**	1569	78	4.97%	1.27	0.92-1.75	0.147	1.22	0.87-1.71	0.253
	**2014**	1588	75	4.72%	1.16	0.84-1.61	0.358	1.14	0.81-1.59	0.461
	**2015**	1484	65	4.38%	1.05	0.75-1.47	0.773	1.05	0.74-1.49	0.796
	**2016**	1594	72	4.52%	1.10	0.79-1.52	0.580	1.11	0.79-1.55	0.541
	**2017**	1934	81	4.19%	1.00			1.00		
**32-36**	**2009**	1483	298	20.09%	1.00	0.84-1.19	0.986	0.93	0.77-1.11	0.405
	**2010**	1531	275	17.96%	0.88	0.74-1.05	0.150	0.82	0.68-0.98	0.029
	**2011**	1601	299	18.68%	0.92	0.78-1.10	0.363	0.86	0.72-1.03	0.091
	**2012**	1662	362	21.78%	1.13	0.96-1.33	0.149	1.07	0.90-1.27	0.452
	**2013**	1569	369	23.52%	1.26	1.07-1.48	0.006	1.19	1.00-1.42	0.048
	**2014**	1588	339	21.35%	1.10	0.94-1.30	0.242	1.04	0.87-1.24	0.678
	**2015**	1484	306	20.62%	1.04	0.88-1.23	0.667	0.97	0.81-1.17	0.771
	**2016**	1594	320	20.08%	1.02	0.87-1.21	0.791	0.98	0.83-1.17	0.846
	**2017**	1934	386	19.96%	1.00			1.00		

Adjusted nominal logistic regression model included maternal characteristics known at the time of the first antenatal visit. Adjustments included maternal age (<20 or ≥35 years), maternal ethnicity (Caucasian, Indigenous and other ethnicities), smoking during pregnancy, low socioeconomic status, pre-existing diabetes, pre-existing hypertension, asthma, pre-existing other maternal conditions, *in vitro* fertilization, history of stillbirth(s), history of PTB and caesarean section in the preceding pregnancy.

OR=unadjusted odds ratio; aOR=adjusted odds ratio; CI=confidence interval, N=number of births, n=number of preterm births, (%) = PTB incidence rate

OR significantly lower than in 2017; OR significantly higher than in 2017
