# Supplementary material for: The elements of success in a comprehensive state-wide program to safely reduce the rate of preterm birth
Source: PLoS One. 2020 Jun 4;15(6):e0234033. doi: 10.1371/journal.pone.0234033 (PMC7272053; doi:10.1371/journal.pone.0234033)
Supplement: S12 Table — (PDF) [file pone.0234033.s012.pdf]

Table S12. Gestational age specific risk of preterm birth in high risk singleton pregnancies in secondary/primary level centers, in unadjusted and adjusted models.

		N	n	(%)	OR	95% CI	p	aOR	95% CI	p
**20-27**	**2009**	4176	16	0.38%	1.09	0.54-2.17	0.817	1.10	0.54-2.24	0.80
	**2010**	3978	16	0.40%	1.15	0.57-2.30	0.699	1.18	0.58-2.41	0.65
	**2011**	4267	20	0.47%	1.33	0.69-2.57	0.394	1.42	0.72-2.80	0.31
	**2012**	4579	19	0.41%	1.20	0.62-2.33	0.596	1.35	0.68-2.67	0.39
	**2013**	4692	17	0.36%	1.04	0.53-2.07	0.904	1.16	0.58-2.33	0.67
	**2014**	4949	15	0.30%	0.87	0.43-1.77	0.709	0.96	0.47-1.96	0.90
	**2015**	4430	9	0.20%	0.59	0.26-1.33	0.202	0.64	0.28-1.47	0.29
	**2016**	4641	17	0.37%	1.07	0.54-2.12	0.845	1.17	0.58-2.33	0.66
	**2017**	4698	16	0.34%				1.00		
**28-31**	**2009**	4176	13	0.31%	1.57	0.67-3.67	0.301	1.46	0.61-3.50	0.39
	**2010**	3978	8	0.20%	1.02	0.39-2.65	0.968	0.98	0.37-2.61	0.97
	**2011**	4267	8	0.19%	0.95	0.37-2.46	0.911	0.95	0.36-2.51	0.91
	**2012**	4579	15	0.33%	1.68	0.74-3.85	0.219	1.74	0.75-4.06	0.19
	**2013**	4692	11	0.23%	1.20	0.50-2.90	0.686	1.25	0.51-3.07	0.62
	**2014**	4949	12	0.24%	1.24	0.52-2.95	0.622	1.29	0.54-3.10	0.56
	**2015**	4430	10	0.23%	1.16	0.47-2.86	0.747	1.20	0.48-2.98	0.69
	**2016**	4641	19	0.41%	2.13	0.96-4.71	0.063	2.23	1.00-4.96	0.05
	**2017**	4698	9	0.19%	1.00			1.00		
**32-36**	**2009**	4176	262	6.27%	0.62	0.53-0.73	0.000	0.62	0.53-0.73	0.00
	**2010**	3978	278	6.99%	0.70	0.60-0.82	0.000	0.69	0.59-0.81	0.00
	**2011**	4267	283	6.63%	0.66	0.57-0.77	0.000	0.64	0.55-0.75	0.00
	**2012**	4579	365	7.97%	0.81	0.70-0.93	0.003	0.78	0.67-0.91	0.00
	**2013**	4692	369	7.86%	0.79	0.69-0.92	0.002	0.77	0.67-0.90	0.00
	**2014**	4949	400	8.08%	0.82	0.71-0.94	0.005	0.80	0.70-0.92	0.00
	**2015**	4430	372	8.40%	0.85	0.74-0.98	0.026	0.82	0.71-0.95	0.00
	**2016**	4641	421	9.07%	0.93	0.81-1.07	0.294	0.90	0.78-1.03	0.12
	**2017**	4698	457	9.73%	1.00			1.00		

Adjusted nominal logistic regression model included maternal characteristics known at the time of the first antenatal visit. Adjustments included maternal age (<20 or ≥35 years), maternal ethnicity (Caucasian, Indigenous and other ethnicities), smoking during pregnancy, low socioeconomic status, pre-existing diabetes, pre-existing hypertension, asthma, pre-existing other maternal conditions, *in vitro* fertilization, history of stillbirth(s), history of PTB and caesarean section in the preceding pregnancy.

OR=unadjusted odds ratio; aOR=adjusted odds ratio; CI=confidence interval, N=number of births, n=number of preterm births, (%) = PTB incidence rate; OR significantly lower than in 2017
