# Supplementary material for: The elements of success in a comprehensive state-wide program to safely reduce the rate of preterm birth
Source: PLoS One. 2020 Jun 4;15(6):e0234033. doi: 10.1371/journal.pone.0234033 (PMC7272053; doi:10.1371/journal.pone.0234033)
Supplement: S13 Table — (PDF) [file pone.0234033.s013.pdf]

Table S13. Gestational age specific risk of preterm birth in high risk singleton pregnancies state-wide in unadjusted and adjusted models.

		N	n	(%)	OR	95% CI	p	aOR	95% CI	p
**20-27**	**2009**	5659	59	1.04%	1.05	0.74-1.48	0.800	1.21	0.84-1.74	0.306
	**2010**	5509	80	1.45%	1.46	1.06-2.03	0.021	1.66	1.19-2.33	0.003
	**2011**	5868	72	1.23%	1.24	0.89-1.72	0.212	1.40	0.99-1.98	0.054
	**2012**	6241	63	1.01%	1.04	0.74-1.46	0.833	1.17	0.82-1.67	0.380
	**2013**	6261	65	1.04%	1.07	0.76-1.50	0.705	1.18	0.83-1.68	0.353
	**2014**	6550	64	0.98%	1.00	0.71-1.41	0.989	1.11	0.78-1.57	0.572
	**2015**	6268	44	0.70%	0.72	0.49-1.05	0.087	0.79	0.53-1.16	0.220
	**2016**	6729	75	1.11%	1.15	0.83-1.60	0.404	1.23	0.88-1.72	0.220
	**2017**	7183	69	0.96%	1.00			1.00		
**28-31**	**2009**	5659	65	1.15%	0.81	0.59-1.11	0.195	0.82	0.59-1.14	0.236
	**2010**	5509	56	1.02%	0.72	0.52-1.00	0.053	0.72	0.52-1.01	0.060
	**2011**	5868	73	1.24%	0.88	0.65-1.20	0.420	0.88	0.64-1.21	0.430
	**2012**	6241	95	1.52%	1.10	0.83-1.46	0.505	1.09	0.82-1.47	0.551
	**2013**	6261	89	1.42%	1.03	0.77-1.38	0.843	1.01	0.75-1.35	0.966
	**2014**	6550	87	1.33%	0.96	0.71-1.28	0.756	0.95	0.71-1.28	0.728
	**2015**	6268	79	1.26%	0.91	0.67-1.22	0.522	0.89	0.66-1.21	0.462
	**2016**	6729	94	1.40%	1.02	0.76-1.35	0.918	1.01	0.76-1.35	0.940
	**2017**	7183	98	1.36%	1.00			1.00		
**32-36**	**2009**	5659	56	9.90%	0.74	0.66-0.82	0.000	0.73	0.65-0.82	0.000
	**2010**	5509	55	10.04%	0.75	0.67-0.84	0.000	0.73	0.65-0.82	0.000
	**2011**	5868	58	9.92%	0.74	0.66-0.83	0.000	0.72	0.64-0.80	0.000
	**2012**	6241	72	11.65%	0.89	0.80-0.99	0.027	0.85	0.76-0.95	0.003
	**2013**	6261	73	11.79%	0.90	0.81-1.00	0.047	0.86	0.78-0.96	0.006
	**2014**	6550	74	11.30%	0.86	0.77-0.95	0.003	0.82	0.74-0.91	0.000
	**2015**	6268	73	11.73%	0.89	0.80-0.99	0.028	0.85	0.76-0.94	0.002
	**2016**	6729	80	12.01%	0.92	0.83-1.02	0.107	0.89	0.80-0.98	0.020
	**2017**	7183	92	12.93%	1.00			1.00		

Adjusted nominal logistic regression model included maternal characteristics known at the time of the first antenatal visit. Adjustments included maternal age (<20 or ≥35 years), maternal ethnicity (Caucasian, Indigenous and other ethnicities), smoking during pregnancy, low socioeconomic status, pre-existing diabetes, pre-existing hypertension, asthma, pre-existing other maternal conditions, *in vitro* fertilization, history of stillbirth(s), history of PTB and caesarean section in the preceding pregnancy.

OR=unadjusted odds ratio; aOR=adjusted odds ratio; CI=confidence interval, N=number of births, n=number of preterm births, (%) = PTB incidence rate

OR significantly lower than in 2017; OR significantly higher than in 2017
