# Supplementary material for: The elements of success in a comprehensive state-wide program to safely reduce the rate of preterm birth
Source: PLoS One. 2020 Jun 4;15(6):e0234033. doi: 10.1371/journal.pone.0234033 (PMC7272053; doi:10.1371/journal.pone.0234033)
Supplement: S14 Table — (PDF) [file pone.0234033.s014.pdf]

Table S14. Stillbirth by year table adjusted for maternal risk factors and pregnancy complications associated with stillbirth.

Year	N	n	Rate/ 1,000	OR	95% CI	p	aOR	95% CI	p
**Established tertiary**									
**2009**	5413	80	14.8	1.15	0.84-1.59	0.385	1.03	0.73-1.45	0.872
**2010**	5510	67	12.2	0.95	0.68-1.33	0.751	0.83	0.56-1.18	0.308
**2011**	5405	75	13.9	1.08	0.78-1.50	0.636	0.95	0.67-1.33	0.749
**2012**	5663	82	14.5	1.13	0.82-1.56	0.455	0.98	0.70-1.37	0.901
**2013**	5452	64	11.7	0.91	0.65-1.29	0.604	0.76	0.53-1.08	0.126
**2014**	5476	74	13.5	1.05	0.76-1.46	0.755	1.03	0.73-1.45	0.884
**2015**	5319	71	13.3	1.04	0.75-1.45	0.814	1.14	0.81-1.61	0.460
**2016**	5304	57	10.7	0.84	0.59-1.19	0.317	0.90	0.62-1.29	0.557
**2017**	5455	70	12.8	1.00			1.00		
**Secondary/primary centres**									
**2009**	24820	73	2.9	0.94	0.68-1.30	0.714	0.99	0.72-1.38	0.973
**2010**	24846	74	3.0	0.95	0.69-1.31	0.772	1.02	0.74-1.41	0.909
**2011**	25821	108	4.2	1.34	1.00-1.80	0.049	1.47	1.09-1.98	0.011
**2012**	27208	78	2.9	0.92	0.67-1.26	0.594	1.01	0.74-1.39	0.941
**2013**	27945	63	2.3	0.72	0.52-1.01	0.054	0.85	0.61-1.19	0.339
**2014**	28525	83	2.9	0.93	0.68-1.27	0.655	1.08	0.79-1.48	0.614
**2015**	26429	69	2.6	0.84	0.60-1.16	0.279	0.98	0.71-1.36	0.911
**2016**	26823	87	3.3	1.04	0.77-1.41	0.806	1.13	0.83-1.53	0.458
**2017**	24987	78	3.1	1.00			1.00		
**State overall**									
**2009**	30233	153	5.1	1.07	1.07-1.34	0.545	1.06	0.84-1.33	0.635
**2010**	30356	141	4.6	0.98	0.98-1.23	0.882	0.99	0.78-1.25	0.913
**2011**	31226	183	5.9	1.24	1.24-1.54	0.047	1.28	1.03-1.59	0.029
**2012**	32871	160	4.9	1.03	1.03-1.28	0.791	1.07	0.85-1.33	0.586
**2013**	33397	127	3.8	0.80	0.80-1.02	0.068	0.87	0.68-1.10	0.244
**2014**	34115	157	4.6	0.97	0.97-1.22	0.814	1.09	0.87-1.37	0.445
**2015**	33944	151	4.4	0.94	0.94-1.18	0.595	1.10	0.88-1.38	0.404
**2016**	34854	150	4.3	0.91	0.91-1.14	0.411	0.98	0.78-1.23	0.842
**2017**	33437	158	4.7	1.00			1.00		

8032 births (27 stillbirths) from the evolving tertiary center were excluded.

OR=unadjusted odds ratio; aOR=adjusted odds ratio; CI=confidence interval, N=number of births, n=number of stillbirths; OR significantly higher than in 2017;

Stillbirth rates are compared using logistic regression analysis with year 2017 as a reference, univariately and after adjustment for maternal age≥35, nulliparity, grand-multiparty, ethnicity, smoking during pregnancy, maternal asthma, low socioeconomic status, history of stillbirth, placental abruption, antepartum haemorrhage for reasons other than placental abruptions and placenta praevia, gestational diabetes and ‘other’ pregnancy complications.
