# Supplementary material for: The elements of success in a comprehensive state-wide program to safely reduce the rate of preterm birth
Source: PLoS One. 2020 Jun 4;15(6):e0234033. doi: 10.1371/journal.pone.0234033 (PMC7272053; doi:10.1371/journal.pone.0234033)

Figure S1. Preterm birth rates between 2013 and 2017 in high risk pregnancies stratified by hospital level and the state overall.

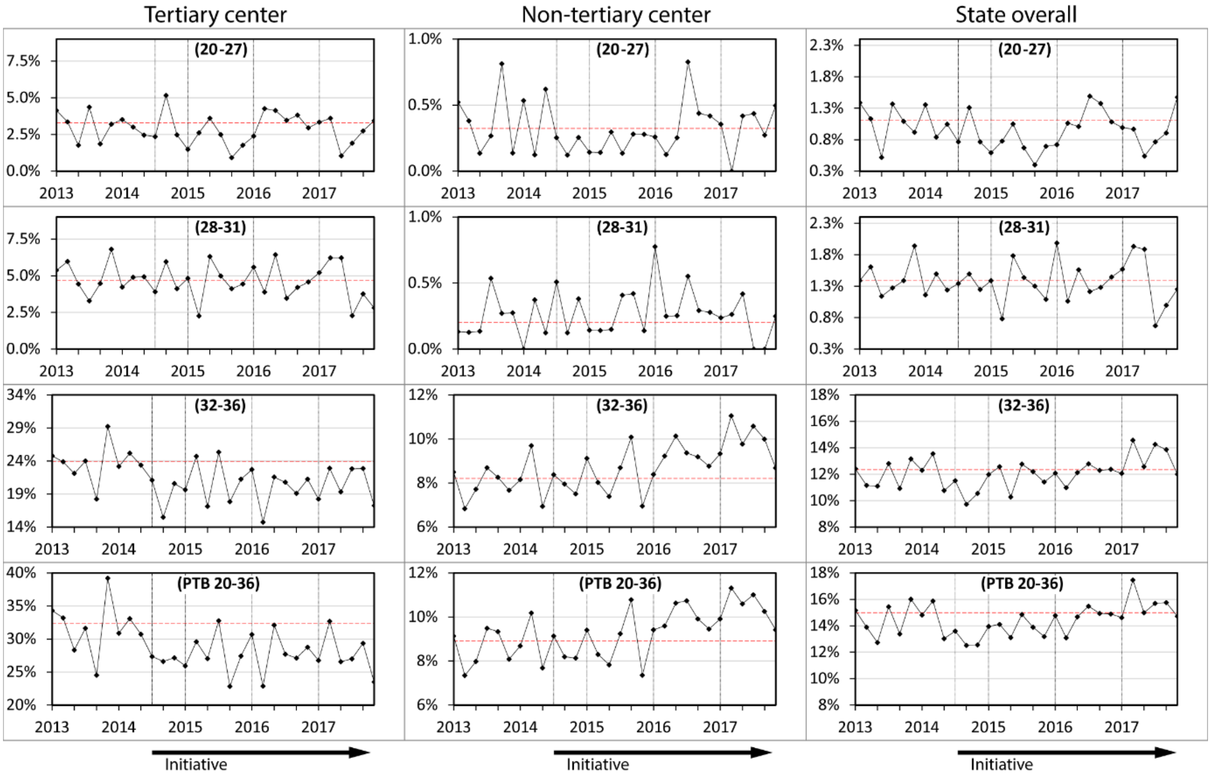

Supplement: S1 Fig — (PDF) [file pone.0234033.s015.pdf]
